# Spiny lobster sounds can be detectable over kilometres underwater

**DOI:** 10.1038/s41598-020-64830-7

**Published:** 2020-05-21

**Authors:** Youenn Jézéquel, Laurent Chauvaud, Julien Bonnel

**Affiliations:** 1 Laboratoire des Sciences de l’Environnement Marin (LEMAR), UMR 6539 CNRS, UBO, IRD, Ifremer, Institut Universitaire Européen de la Mer (IUEM), LIA BeBEST, rue Dumont D’Urville, 29280 Plouzané, France; 2 Woods Hole Oceanographic Institution, Applied Ocean Physics and Engineering Department, Woods Hole, MA 02543 USA

**Keywords:** Ecology, Conservation biology

## Abstract

The detection ranges of broadband sounds produced by marine invertebrates are not known. To address this deficiency, a linear array of hydrophones was built in a shallow water area to experimentally investigate the propagation features of the sounds from various sizes of European spiny lobsters (*Palinurus elephas*), recorded between 0.5 and 100 m from the animals. The peak-to-peak source levels (SL, measured at one meter from the animals) varied significantly with body size, the largest spiny lobsters producing SL up to 167 dB re 1 µPa^2^. The sound propagation and its attenuation with the distance were quantified using the array. This permitted estimation of the detection ranges of spiny lobster sounds. Under the high ambient noise conditions recorded in this study, the sounds propagated between 5 and 410 m for the smallest and largest spiny lobsters, respectively. Considering lower ambient noise levels and different realistic propagation conditions, spiny lobster sounds can be detectable up to several kilometres away from the animals, with sounds from the largest individuals propagating over 3 km. Our results demonstrate that sounds produced by *P. elephas* can be utilized in passive acoustic programs to monitor and survey this vulnerable species at kilometre scale in coastal waters.

## Introduction

Passive acoustic monitoring (PAM) of marine species has recently gained attention by biologists and is now used worldwide. This is due to the increased knowledge of animal sound repertoires, and the behavioral contexts in which they are produced^1–3^. In addition, the density of seawater enables sounds to propagate over greater distances compared to air^4^. Estimating the detection ranges between a particular sound-producing species and a receiver can give crucial information about its spatial distribution in an ecosystem. These calculations rely on the measurements of the source level (SL, *i.e*. the sound pressure level recorded at 1 m from the source) and the transmission loss (TL, *i.e*. the attenuation of the sound as it propagates away from the source) of animal sounds underwater. For example, marine mammal sounds can be detected kilometres away in shallow and deep oceans with hydrophones^5–7^. Fish also produce sounds in shallow waters that can be detectable from few meters^8,9^ to hundreds of meters away^10,11^. However, data available on sound propagation and detection ranges for crustaceans are scarce, though crustaceans are known to emit a large diversity of sounds^12–14^.

Marine arthropods produce sounds that are mostly characterized by broadband pulses, *i.e*. short transient sounds with a large bandwidth^15–17^. Estimating their sound propagation may be challenging as they inhabit shallow coastal waters (at depths below tens of meters). This implies complex sound fields due to physical constraints such as the presence of boundaries created by the water surface and the seabed^18,19^, and it is thus difficult to accurately model sound propagation^20^. Until now, detection ranges of crustacean sounds have relied on crude estimations of SLs performed using distant measurements that are then artificially back-propagated to 1 m by using theoretical propagation models^21,22^. In addition, some studies have been performed in tanks. Tank experiments are very convenient since distances between receivers and animals can be precisely measured^23^. However, tank acoustics are complicated. The relatively small volumes and close boundaries of tanks highly affect sound propagation as well as SL estimates^13,14^; these tank effects have been largely ignored by most previous studies (as a counter example, see^15^ for an experimental illustration on the differences of crustacean sounds recorded in tanks and *in situ*). Thus, there is a need to combine theory with empirical measurements of site-specific sound propagation to obtain reliable SL and TL predictions for estimating detection ranges of crustacean sounds. For this purpose, arrays of hydrophones are useful because they can accurately estimate SL and TL in shallow waters^11,24^.

Estimating SL requires an accurate knowledge of the distances between sound producers and receivers. This problem is likely easier for many benthic crustaceans than for marine mammals and fish, since they have a relatively low mobility. Spiny lobsters are particularly good models for performing such studies because they produce specific sounds termed “antennal rasps”. While these antennal rasps are characterized by trains of broadband pulses with sound intensity spread over a wide bandwidth, their spectrums are dominated by low frequencies (i.e. below 1 kHz^15,25^). In addition, it is possible to induce sound production by handling the animals underwater (which aims to imitate a predator attacking^15,16,25^). Hence, these animals are practical for accurately measuring SL and propagation of produced sounds over different distances, while precisely controlling their positions. Interestingly, sounds produced by spiny lobsters have similarities with insects^26^. Several studies in terrestrial arthropods have shown that the intensity of their sounds depends on body size^27,28^. If such a relation also exists in spiny lobsters, this implies that larger individuals likely produce higher amplitude sounds compared to smaller animals. As a result, these large individuals should be detectable over longer distances. In addition, a recent study^15^ recorded antennal rasps from the European spiny lobster (*Palinurus elephas*) underwater and reported high peak-to-peak sound pressure levels (SPL) up to 170 dB re 1 µPa^2^ at 20 cm from the animals. Such elevated SPLs imply these sounds could be detectable during *in situ* PAM studies, which is needed to better manage this highly commercially valuable and vulnerable species that has become scarce in European coastal waters due to overfishing^29,30^. However, it is now crucial to understand the variability of their sounds (*i.e*. with animal size), how they propagate and at what distances they can be detectable underwater with hydrophones before PAM can be used operationally to monitor spiny lobsters.

In this context, the aim of this study was to provide new insights on the propagation features of broadband sounds produced by a marine crustacean, the European spiny lobster (*P. elephas*), in a shallow coastal water area. First, we measured the SPLs of individuals from various sizes (from 2.6 to 13.5 cm of carapace length) using a linear array of 8 hydrophones placed between 0.5 and 100 m from the animals. Using this set-up, the SLs, in terms of SPLs, were obtained at 1 m from the animals. Secondly, we estimated the associated TLs using a simple model *a*×log_10_(r), with r being the source-receiver distance and *a* being the model parameter to be calculated, and we compared the estimated TLs with theoretical models. Based on these results, the detection ranges (and their variability with animal size) were estimated using different conditions of ambient noise levels (ANL) and TL models. Lastly, we examined changes in the spectral contents of the antennal rasps with increasing distance from the spiny lobsters, and discussed their potential ecological implications.

## Results

During the experiment, the wind state ranged between 0 (calm) and 2 (light breeze) on the Beaufort scale, corresponding to speeds between 1 and 6 knots. Seawater temperature was 15.3 °C and salinity was 35. The water depth was 9 m. Anthropogenic noise from a near marina contributed to the low frequencies (below 1 kHz) in the recorded ambient noise, and was mainly produced by ship motor noise and the chains of the boats’ buoys rubbing against the bottom. However, the ANL in the frequency band from 10–78 kHz was quieter compared to the ANL calculated over the entire (0.001–78 kHz) frequency band (over 20 dB difference), and varied slightly across the different hydrophones with a mean of 88 ± 4 dB re 1 µPa^2^. The sources of ambient noise in the frequency band 10–78 kHz were attributed to isolated broadband pulses from unknown sources.

**Figure 1:**
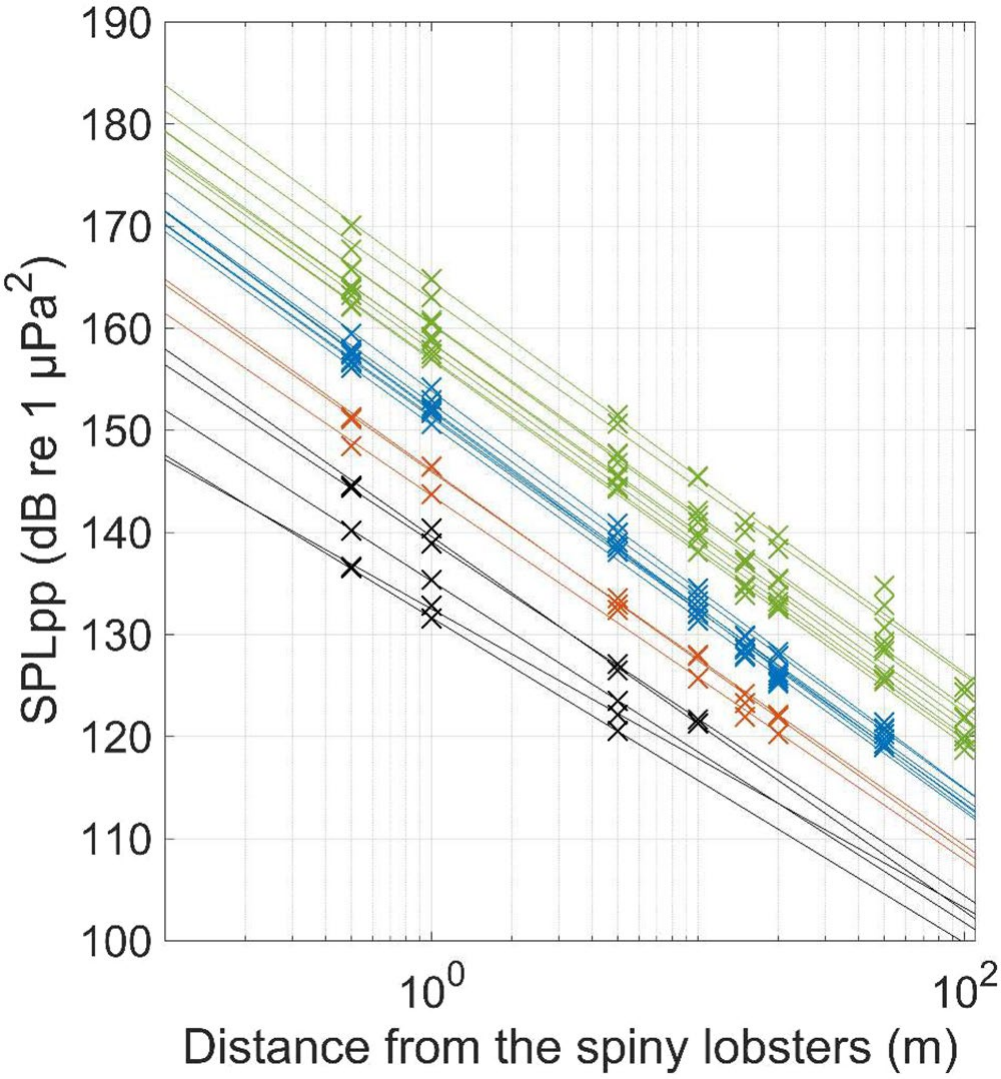
Evolution of sound pressure levels in peak-to-peak (SPL_pp_; crosses) recorded at different distances from the spiny lobsters (between 0.5 and 100 m) and their calculated fit curves using logarithmic models (continuous lines). Each point represents the mean SPL_pp_ averaged on 10 sounds. The x-axis is in logarithmic scale. Each value calculated at 1 m corresponds to the source level (SL_pp_) of the spiny lobsters. The colors are related to the body size of the spiny lobsters (green: large, blue: intermediate, orange: small, black = very small).

We extracted and analyzed manually a total of 1560 antennal rasps from the sound recordings of spiny lobsters (N = 24) with the linear array of 8 hydrophones placed between 0.5 and 100 m. This total number of analyzed antennal rasps corresponds to the 1920 antennal rasps that were recorded (10 per animal and per recording distance), minus 360 antennal rasps that were not analyzed because the signal-to-noise ratio was too low (i.e. the antennal rasps were buried in the ambient noise). Indeed, antennal rasps from intermediate (N = 7), small (N = 3) and very small (N = 5) individuals were not recorded beyond 50, 20 and 5 m, respectively (see Fig. 1). In marked contrast, all sounds from large spiny lobsters (N = 9) were recorded on all the hydrophones, with distance up to 100 m.

The SL_pp_ varied significantly and positively according to the body size of spiny lobsters (Pearson: r = 0.910, t = −10.316, N = 24, df = 22, p < 0.001). Indeed, we calculated a maximum difference of 35 dB between the smallest (SL_pp_ = 132 dB re 1 µPa²; CL = 2.6 cm) and the largest (167 dB re 1µPa²; CL = 13.5 cm) spiny lobsters (Fig. 1). The maximum calculated SPL_pp_ was 172 dB re 1 µPa^2^ and was produced by the largest individual (CL = 13.5 cm) at 0.5 m.

**Figure 2:**
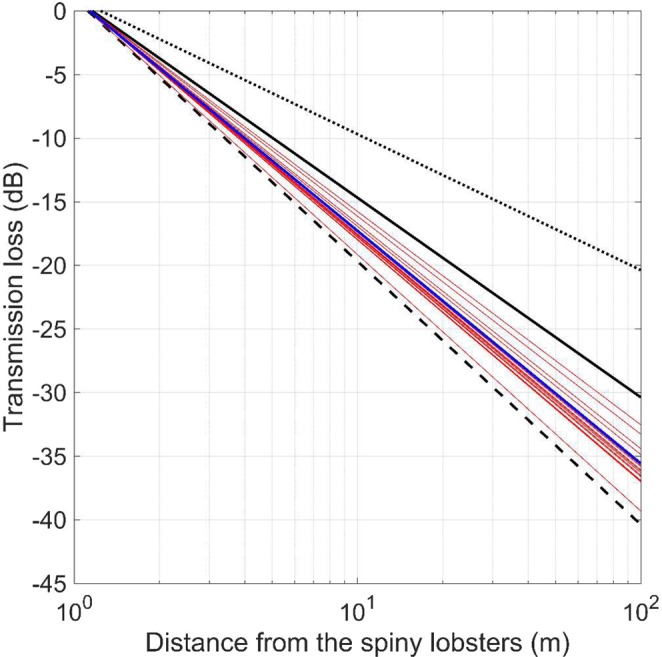
Transmission losses (TL) estimated for 19 spiny lobsters (red lines) based on the dataset SPL_rms_ vs. distance, and the global fitted TL (blue line, *a =* 17.6). Black lines represent theoretical models of TL: dotted is cylindrical (*a* =10), continuous is practical (*a* = 15), dashed is spherical (*a* = 20).

The TL models estimated as *a*×log_10_(r) from the dataset of SPL_rms_ vs. distance did not significantly vary with body size (Pearson: r = −0.175, t = −0.733, N = 19, df = 17, p = 0.474), as expected. The estimated TL parameter *a* ranged between 16.1 and 19.5 (Fig. 2). By fitting the results amongst all individuals (except the very small ones), the global TL parameter *a* was 17.6 which is between the theoretical models of practical (*a* = 15) and spherical (*a* = 20) TLs (Fig. 2).

Detection ranges of the antennal rasps produced by all spiny lobsters were estimated per group of body size by considering the calculated SL_rms_ and global TL (*a* = 17.6). Their values are summarized in Table 1. Under the ANL conditions encountered during the experiment, we estimated that large individuals can be recorded up to 250 m, with the largest individual (CL = 13.5 cm) being detectable up to 410 m (Table 1). Interestingly, the estimated detection ranges for smaller individuals are less than 100 m, and are thus covered by the range of our array. Thus, these estimated values are consistent with the values observed on the *in situ* recordings. Indeed, intermediate, small and very small spiny lobsters could not be recorded at 100, 50 and 10 m (respectively; see Fig. 1), which matches our estimations of detection ranges (Table 1). In a theoretical low (but still realistic) ANL, large individuals can be detectable at 750 m, with the largest individuals (CL = 13.5 cm) being recorded up to 1080 m. By considering a practical loss model for TL (the most realistic), large spiny lobsters could be detectable at the kilometre scale under the theoretical ANL, with largest individuals being detected up to 1740 m (Table 1). Using an attenuated cylindrical loss model for TL (the least conservative), all spiny lobsters (except the very small ones) may be detectable at the kilometre scale under the theoretical low ANL, with largest spiny lobsters being detected up to 3610 m (Table 1).

**Table 1:** Estimations of detection ranges (in m) of antennal rasps produced by spiny lobsters underwater. The averaged values are reported per group of size-matched spiny lobsters (see Material and Methods for details). The different transmission losses (TL) used correspond to the global TL (*a* = 17.6) calculated between all spiny lobsters (except the very small ones), and the theoretical models of cylindrical TL (*a* = 10) and practical (*a* = 15) TLs. Min is signal-to-noise ratio (SNR) = 5 dB and max is SNR = 10 dB.

	TL	Wenz 5 knots		This study	
		Min	Max	Min	Max
		ANL = 81	ANL = 86	ANL = 93	ANL = 98
Large (N = 9)	17.6	750	500	250	150
	15	1330	940	510	300
	10	3040	2450	1690	1180
Intermediate (N = 7)	17.6	390	230	100	60
	15	850	550	270	140
	10	2250	1710	1030	620
Small (N = 3)	17.6	210	120	50	27
	15	420	240	100	50
	10	1510	1030	500	240
Very small (N = 5)	17.6	70	40	15	8
	15	130	70	25	10
	10	620	330	100	40

**Figure 3:**
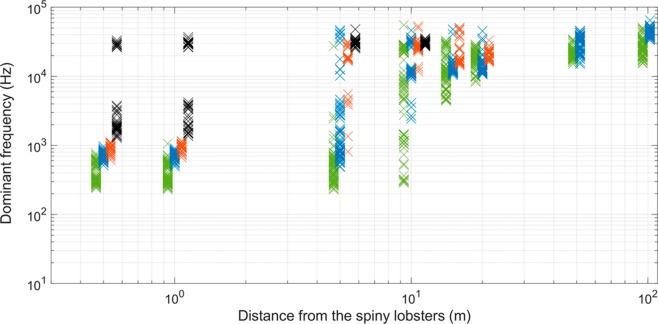
Dominant frequencies calculated on the recorded antennal rasps as a function of the animal-hydrophone distance. Each point represents the value from one antennal rasp. The colors are related to the body size of the spiny lobsters (green: large, blue: intermediate, orange: small, black: very small). Both x- and y-axis are in logarithmic scale.

**Figure 4:**
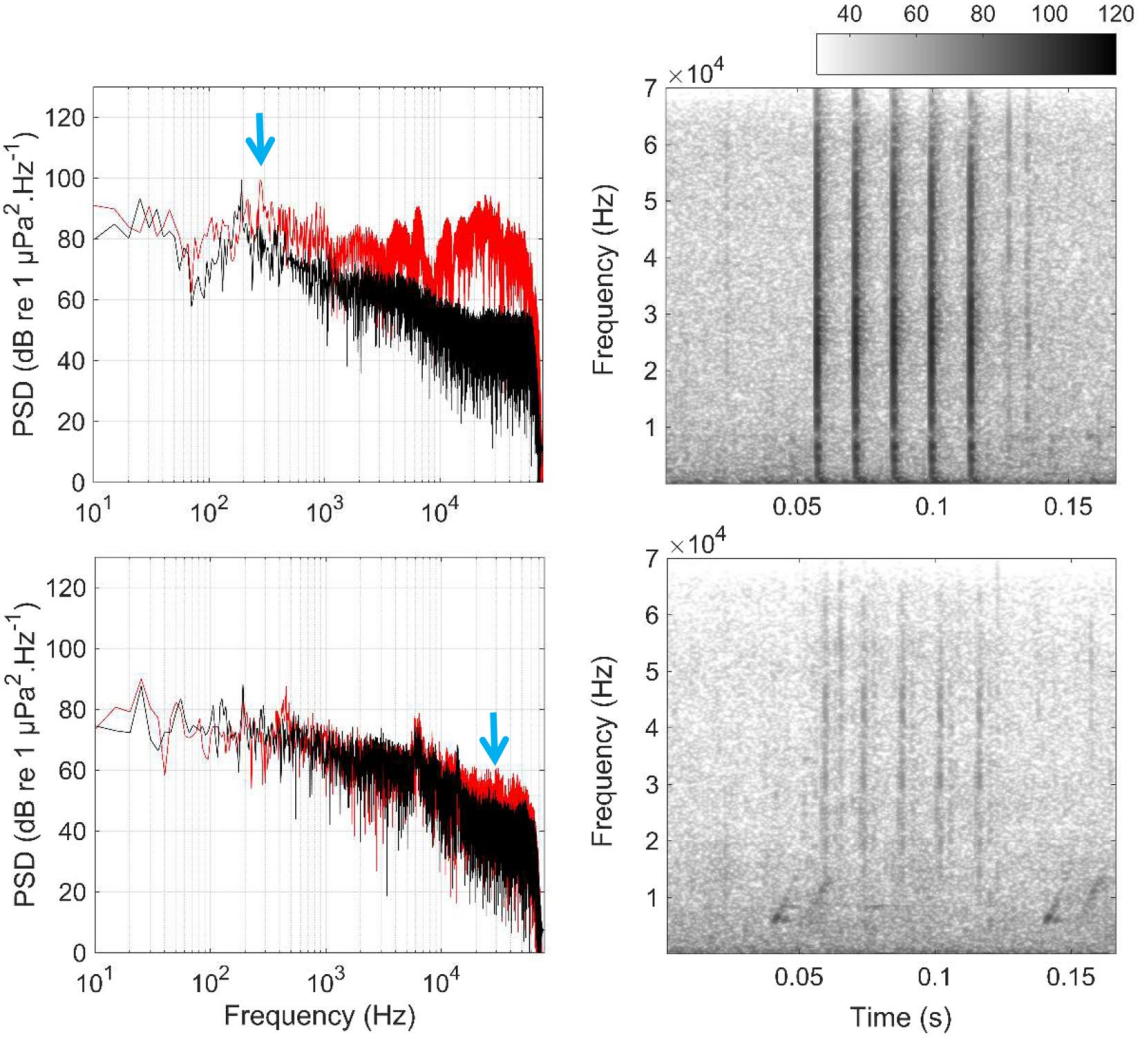
Power spectral densities (PSD, left) and spectrograms (right) of an antennal rasp produced by a large spiny lobster (carapace length = 13 cm) at 1 m (top) and 100 m (bottom). The black lines are the ambient noise recorded at the same distances. The blue arrows show the dominant frequencies of the recorded antennal rasp, calculated at 0.3 kHz at 1 m while it was calculated at 30 kHz at 100 m. The color scale bar is in dB re 1 µPa^2^.Hz^−1^.

Overall, a clear pattern occurred in changes of the spectral content of recorded antennal rasps with increasing distance. The spectral content was dominated by low frequencies (<1 kHz) close to the spiny lobsters (<10 m) whereas only high frequencies (>10 kHz) remained far from the animals (>10 m; Figs. 3 and 4). However, antennal rasps produced by very small spiny lobsters did not present any low frequency content (<1 kHz) even at 0.5 m (range 1.3 to 31.3 kHz). The low frequency content was probably masked by the ambient noise due to their low intensity features. Above 10 m, all recorded antennal rasps had dominant frequencies only between 10 and 60 kHz (Figs. 3 and 4).

## Discussion

### Passive acoustic monitoring

In the bioacoustic literature, only one study used passive acoustics to monitor spiny lobsters (*Panulirus japonicus*) underwater^31^. The authors found an increase in the production of antennal rasps on the nights of large tidal changes, which complement the ecological knowledge on the nocturnal behaviour of spiny lobsters^32^. However, the propagation features of the recorded antennal rasps were not assessed, and thus the detection ranges at which spiny lobsters produced sounds were not quantified, which drastically limits the impacts of the corresponding study. To fill this gap, the present paper is the first to experimentally examine the propagation features of the antennal rasps produced by the European spiny lobster (*P. elephas*) in shallow waters using a linear array of hydrophones with a range of 100 m.

Although SL_pp_ values have been reported for several marine species of mammals and fish, this is the first time that SL_pp_ values for a marine crustacean are reported from direct *in situ* recordings. Spiny lobsters produce loud antennal rasps, with SL_pp_ up to 167 dB re 1 µPa^2^ for the largest individuals (CL = 13.5 cm; Fig. 1). Overall, our results confirm the range of values obtained by a recent study^15^ with the same species where SPL_pp_ (calculated only at 20 cm from the source) were calculated above 170 dB re 1 µPa^2^. The antennal rasps produced by spiny lobsters have exceptionally elevated SL_pp_ among crustaceans. Indeed, only snapping shrimps have been reported to produce higher SL_pp_^12,17^, estimated in tanks up to 215 dB re 1 µPa^2^. We also found that SL_pp_ of spiny lobsters vary significantly and positively with their body sizes, with larger individuals producing higher amplitude sounds compared to smaller individuals. Indeed, the smallest individuals (CL = 2.6 cm) had SL_pp_ calculated at 132 dB re 1 µPa^2^, which was 35 dB less compared to the largest individuals (CL = 13.5 cm; Fig. 1). These variations in sound intensity with body size have already been described in insects whose sound production mechanisms are similar to spiny lobsters^26–28^. To our knowledge, no studies have examined ontogenetic variations of sound intensity in crustaceans. Further studies should relate these variations with the development of sound-producing structures in spiny lobsters.

The use of linear arrays of hydrophones is known to be useful to estimate SL and TL for marine mammals and fish^11,24^. In marked contrast to these highly mobile animals, spiny lobsters produce sounds while handling, which enables precise control of their distances from the recording hydrophones. This allowed us to perform accurate measurements of the SPL_pp_ and SPL_rms_ over distance from the spiny lobsters (between 0.5 and 100 m; Fig. 1). While we found that SL_pp_ (and SL_rms_) varied with body size, we did not find any significant relationship between the different coefficients of TL and the body sizes of the spiny lobsters. This result was to be expected: the physics of sound propagation does not depend on the sound source, and it is thus independent from the animal body size. The estimated TL [17.6×log_10_(r)] is consistent with sound propagation in shallow water^18–20^, but different from simplistic models (e.g. cylindrical losses) that are often used in bioacoustics. This clearly demonstrates the importance of correctly assessing sound propagation in shallow waters to study crustaceans’ sounds. Last but not least, we only recorded sounds produced by spiny lobsters while facing the linear array of hydrophones (i.e. on the same axis). Thus, we did not quantify the potential directivity of their sound source. The use of three-dimension arrays will be useful in further studies to better describe the directivity and 3D propagation of antennal rasps in shallow waters^11^.

An important application of measuring accurately SL and TL is the estimation of detection ranges at which animals can be detectable underwater with hydrophones. We found that the SLs of spiny lobsters varied with body size, and thus their detection ranges too. Indeed, during the sound recordings, only large individuals were recorded with the hydrophone placed at 100 m (Fig. 1). In marked contrast, intermediate, small and very small individuals were not recorded with our hydrophones above 50, 20 and 10 m, respectively (Fig. 1). These results were confirmed through the detection ranges estimated using the passive sonar equation, where intermediate, small and very small individuals can be detectable up to 100, 50 and 15 m, respectively (see Table 1). Under the ANL recorded during the experiment, large individuals are expected to be recorded at 250 m underwater with the largest spiny lobsters (CL = 13.5 cm) being detectable above 400 m (Table 1). Using a theoretical low (but still realistic) ANL^33^, the detection ranges for large individuals were estimated at 750 m with the largest individuals being detectable up to 1080 m. In this study, the global TL calculated for spiny lobsters ranged between the theoretical models of practical and spherical TL, which are the most conservative models of TL (*i.e*. loss of 40 dB at 100 m for the spherical TL). By considering the least conservative model of TL (cylindrical), large spiny lobsters may be detectable at the kilometre scale under the theoretical ANL, with largest individuals being detected up to 3000 m (Table 1). This is consistent with a previous study which recorded broadband sounds likely produced by unknown species of invertebrates at kilometres away from coral reefs^34^. This result is particularly important, because it demonstrates the possibility to use PAM for studying spiny lobsters underwater. Such studies would have a large spatial resolution, which is highly valuable for assessing their absence-presence and activity patterns in their environments, as shown for marine mammals and fish^35,36^.

### Ecological relevance

From an ecological point of view, the detection ranges discussed above may not be relevant for spiny lobsters. A more interesting quantity is the communication distance^9,11^, i.e. the distance over which animals can “hear” each other. Communication distances are more difficult to assess because it requires additional information about the animal hearing abilities. To our knowledge, no audiogram has been performed in spiny lobsters, and nothing is known about their exact hearing sensitivity. However, it is thought that most crustaceans are sensitive to low frequency particle motion (i.e. below 1 kHz)^37,38^. Assuming that this hypothesis holds true for spiny lobsters, our study may be used to roughly estimate the communication distances by evaluating the detection ranges of the low frequencies from antennal rasps. Nonetheless, we emphasize that only a rough estimate can be obtained since (1) we measured only sound pressure, and (2) the hearing capacity of spiny lobsters is not known. The following discussion will thus be qualitative, rather than quantitative.

The spectral content of the recorded antennal rasps was different according to the distance from the spiny lobsters. Indeed, while low frequencies (<1 kHz) dominated close to the sound source (<10 m), only high frequencies (>10 kHz) remained at higher distances (>10 m; Figs. 3 and 4). Since the spiny lobsters may be sensitive to low frequencies only (<1 kHz), the long-distance high-frequency antennal rasps are likely out of the hearing range of the animals. Thus, we can conclude that the communication distances are much shorter than the detection ranges. On the specific dataset studied here, assuming that the animals are sensitive to frequencies below 1 kHz, the communication distances would be no more than 10 m (see Fig. 3), which is consistent with what is known for fish^8,9,11^.

The apparent frequency shift of the antennal rasps is due to sound propagation and ambient noise, and not ecological reasons. Indeed, the ANL in the low frequencies (below 1 kHz) was 20 dB louder compared to the higher frequencies (above 10 kHz). As sounds propagate away from the source, their intensities decrease. Thus, at large ranges (above 10 m), the apparent dominant frequencies (<1 kHz) of the antennal rasps are fully masked by the high-power low-frequency ambient noise. The recorded sounds have therefore dominant frequencies only above 10 kHz, i.e. in the frequency band where the high frequency part of the antennal rasps is not masked by the ambient noise (see Fig. 4). Interestingly, another study^31^ recorded sounds produced by *Panulirus japonicus* and the authors found that the recorded antennal rasps were also dominated by high frequencies (above 10 kHz). This study was performed in a shallow water area at depths similar as our study (between 7 and 11 m). Thus, our results show that the apparent high dominant frequencies observed at large ranges are due to acoustic masking, and likely have no ecological meanings for spiny lobsters.

In this study, we recorded sounds in very shallow waters mainly for practical reasons. However, *P. elephas* is known to inhabit coastal waters from the shore up to depths of 200 m, and large individuals are most commonly found between 50 and 100 m depths^39,40^. In addition, we recorded antennal rasps in a flat sandy bottom, while spiny lobsters tend to live in rocky habitats^29,41^. Overall, detection ranges and communication distances may change depending on the environment both because of sound propagation and ambient noise. We evaluated detection ranges by using different propagation models, and the obtained results ranged between 10 and 3000 m (depending on the size of the animals). Although simple, the models considered here represent reasonable bounds within which more realistic models may predict propagation. It is thus expected that the order of magnitude of the detection ranges evaluated in this paper hold also for other environments. On the other hand, the communication distances have been evaluated without using propagation models. Nonetheless, the estimated values are of the same range as the water depth (i.e. 10 m). Their propagations are thus impacted little by the environment (TLs are usually modeled using simple spherical losses)^42^. Hence, we predict that the order of magnitude of the communication distances would be similar in other environments. This must now be confirmed through the development of audiograms and the measurements of particle motion generated by the antennal rasps underwater^37,43^.

### Towards a better monitoring of a vulnerable and cryptic species

In conclusion, the use of a linear array of hydrophones permitted us to examine for the first time the propagation features of the antennal rasps produced by spiny lobsters in a shallow water area. The accurate measurements of SL and TL allowed us to estimate detection ranges underwater by considering different conditions of ANL. We notably found that sounds from spiny lobsters can be detectable kilometres away underwater with hydrophones. These results will be helpful for further PAM studies because they can allow potential detection, localization and even estimation of densities of *P. elephas* over large spatial and temporal scales underwater^44,45^. The development of such non-invasive and non-destructive tools is needed to better manage this highly commercially valuable and vulnerable species that has become scarce in European coastal waters due to overfishing^29,30^.

## Methods

### Animal collection, characteristics and care

For the experiments, we used a total of 24 *P. elephas* individuals of a wide range of sizes. Only inter-moult individuals with full sets of intact appendages were selected for this study.

We carefully collected 17 juveniles by hand while scuba diving in the Bay of Perros Guirec (48°50′2.044″ N, 3°26′28.312″ W) at depths between 10 and 25 ms during two diving sessions on May 28^th^, 2019. Juvenile individuals (3 males and 14 females) had carapace lengths (CLs) between 2.6 and 8 cm, as measured from the anterior tip of the rostrum to the medial point of the posterior carapace margin. Large adult individuals were bought from local fishermen several days after they were captured in the Iroise Sea on May 21^st^, 2019. These 7 large spiny lobsters (2 males and 5 females) had CLs between 9.5 and 13.5 cm.

After capture, all individuals were immediately transferred to an isolated, quiet room in the facilities of the Institut Universitaire Européen de la Mer (IUEM) in Plouzané (France). They were placed in holding tanks of different dimensions according to their size: three plastic-sided rectangular tanks (0.60 m × 0.50 m × 0.35 m, length × width × effective height; seawater volume = 0.1 m^3^) and two plastic-sided square tanks (1.0 m × 1.0 m × 0.6 m, 0.6 m^3^). There were 4 to 8 individuals per holding tank. Before they were placed in these tanks, all individuals were tagged using alternating small white and black rubber bands placed on the base of their second antennae. Each tagged individual was described (size, sex) and given an identification number.

Holding tanks were continuously supplied with the same sand-filtered seawater pumped from the Bay of Brest. During the holding period, temperature was 14.6 ± 0.6 °C and salinity was 34.7 ± 0.1. All animals were fed with fresh pieces of fish (mackerel) *ad libitum*. They were kept under a 12:12 photoperiod; daylight conditions (from 08:00 am to 08.00 pm) were simulated with fluorescent light tubes placed above the holding tanks. Several sections of rigid, PVC pipes associated with large rocks were provided as shelters in each tank. Animals were acclimatized at least 15 days in these holding conditions before they were used in the at-sea recording experiment.

### Sound recordings and video

A linear array of 8 hydrophones was used to record sounds produced by the spiny lobsters (see Table 2 for characteristics of the recording devices). The 8 hydrophones were spaced relative to the handled spiny lobsters at 0.5, 1, 5, 10, 15, 20, 50 and 100 m. The 2 closest hydrophones (0.5 and 1 m) were set with a gain of 0 dB which permitted us to characterize the powerful antennal rasps without clipping the recorded sounds (*i.e*. sound saturation).

**Table 2:** Characteristics of the recording devices used for the linear array of hydrophones (placed between 0.5 and 100 m from the spiny lobsters) during *in situ* recordings. Fs: frequency sampling.

Hydrophone			Recorder			Distance from the spiny lobsters (m)
Model	Flat frequency response (kHz)	Sensitivity (dB re 1 V µPa^−1^)	Model	Gain (dB)	Fs (kHz)	
HTI-99-HF	0.002–125	−174.9	EA-SDA14 (RTSys, France)	0	156	0.5
		−174.7		0	156	1
		−174.9		15	156	5
		−175		15	156	10
		−174.8	EA-SDA14 (RTSys, France)	15	156	15
		−174.7		15	156	20
HTI-96-MIN	0.002–30	−163.8	Wildlife Acoustics Song Meter (Model SM2)	24	96	50
HTI-92-WB	0.002–50	−155.5	EA-SDA14 (RTSys, France)	15	156	100

Video recordings were made during *in situ* recordings using two GoPro HERO3 cameras at a recording rate of 29.97 frames per second. The videos allowed confirmation of sound production by each spiny lobster tested, and also provided the time at which the handled individuals were placed at the source point during the sound recordings. They also validated the identification of each tested individual by checking the rubber bands on the spiny lobsters’ second antennae.

### Location and characteristics of the experimental site

The experimental site where sound recordings were performed was located in the Bay of Saint Anne de Portzic (48°21'32.951” N, 4°32'59.024” W) in the Bay of Brest, just beneath the facilities of the IUEM where spiny lobsters were held. It was located about 100 m outside a marina hosting 120 recreational boats. It is a shallow water area with depths varying between 15 m during high tide and 9 m during low tide. The bottom is flat and composed of homogenous, fine sand with empty shells.

### Experimental set up

The day prior the recording experiment, while scuba diving, all spiny lobsters were transferred into three galvanized steel cages (1.0 m × 1.0 m × 0.5 m, 0.5 m^3^) placed side by side linearly on the bottom near a rocky dyke. Sections of rigid PVC pipes were provided as shelters. Spiny lobsters were acclimatized for 24 hours in these conditions to recover from transport and handling.

The next day (June 14^th^, 2019), while scuba diving, the linear array of hydrophones was built in front of the center holding cage. First, a rope was laid on the substrate, which was previously marked at each distance where the different hydrophones should be placed. Then, hydrophones were attached 0.5 m above the bottom to metal rods anchored with concrete tubes at each mark placed on the rope. Cables were anchored to the bottom with lead weights and recorders were laid on the bottom. Because the Wildlife recorder had a positive buoyancy, it was anchored to the bottom using a lead weight. Thus, its hydrophone (placed at 50 m from the spiny lobsters) was located at 1 m above the bottom. The two cameras (same model GoPro as mentioned above) were placed on the top of the 2 outside cages, in front of the center cage. Then, the boat transporting scuba divers was anchored 200 m away from the cages, and its motor was shut down. Sound recordings were performed during low tide to avoid tidal currents. Before the recording experiment started, the ambient noise was recorded for 10 minutes without scuba divers underwater. Next, each spiny lobster was gently picked up, handled one by one, and positioned at the source point. The source point, defined as the point where spiny lobsters were recorded, was located at the beginning of the rope, at 0.5 m from the first hydrophone. Each individual was maintained at the same distance above the bottom (0.5 m) as the hydrophones during recordings, and the spiny lobsters were held so that they faced the linear array of hydrophones. Thus, the body of the animals was on the same axis as the linear array of hydrophones. We chose to handle spiny lobsters to elicit their sound production, as this method is commonly described in the bioacoustic literature on spiny lobsters^15,16,25^. Each sound recording for the different spiny lobsters lasted between 20 and 30 s. During each sound recording, the two scuba divers stopped their breath to avoid the emission of intrusive noise related to air bubbles. In total, the recording experiment lasted 60 min. In the end of the recordings, five sharp raps were made on the cage walls which permitted us to synchronize both hydrophones and GoPros.

### Sound analysis

#### Sound features of antennal rasps

Synchronized recordings of sounds (in.wav format) and videos were first analyzed to confirm sound production by each tagged spiny lobster. Then, each antennal rasp was extracted manually using the Audacity software (version 2.1.1^46^). Antennal rasps were defined as pulse trains composed of at least several pulses separated by less than 20 ms from each other. Hence, any isolated pulses present in the recordings were not analyzed here. We performed sound analysis on a total of 10 antennal rasps per spiny lobster and per distance (80 sounds analyzed per individual in total). The same sounds were analyzed at the 8 different distances for each spiny lobster. All sequences were then processed using custom MATLAB scripts (version 9.1; The MathWorks).

We calculated the intensity features of the antennal rasps based on their sound pressure levels (SPL, in dB re 1 µPa^2^) both in peak-to-peak (for biological interpretation) and root-mean-square (for detection range estimations using the passive sonar equation).

As these sounds are pulse trains characterized by short and transient pulses, we first chose to calculate the peak-to-peak SPL (SPL_pp_) which is the most representative and practical intensity feature for these types of biological sounds^15,47^. As we recorded antennal rasps at several distances from the spiny lobsters, the SPL calculated at 1 m was referred to as the source level (SL_pp_). When pulse trains were affected by low frequencies related to ambient noise (below 50 Hz), especially at long distances (50 and 100 m), we measured the SPL_pp_ based on the pulse with the highest and lowest amplitude of the train to avoid overestimating their values. When pulse trains could not be isolated from the ambient noise, we did not calculate their SPL_pp_. We then averaged the SPL_pp_ calculated per distance and per individual for further analysis. The SPL_pp_ and SL_pp_ were used for biological sound characterization. Because these values varied according to the body size of the spiny lobsters (see Results), we chose to regroup the averaged values per group of size-matched individuals for a better overall description. Four different groups of body sizes were defined and termed as follow: large (8.0 <CL < 13.5 cm, N = 9), intermediate (6.4 <CL < 7.3 cm, N = 7), small (4.2 <CL < 4.8 cm, N = 3) and very small (2.6 <CL < 3.1 cm, N = 5).

We also calculated the SPL and SL as root-mean-square (SPL_rms_ and SL_rms_, respectively) by integrating the power spectral density (PSD, in dB re 1 µPa^2^.Hz^−1^) of the antennal rasps between 10 and 78 kHz (bandwidth where intensity from the antennal rasps remained above 10 m from the spiny lobsters, see Results). The SPL_rms_ at 50 m was calculated over the 10–48 kHz frequency band, because the system specification did not allow measurements of frequencies above 48 kHz (see Table 2). Because antennal rasps are characterized by pulse trains, the SPL_rms_ and SL_rms_ values averaged on the entire length of the antennal rasps would be underestimated. We instead chose to calculate them on each pulse inside the pulse trains over a 1 ms window length (Fast-Fourrier Transform size: 156 points) centered on the pulse. Then, each value was averaged over all pulses present in an antennal rasp to obtain its mean SPL_rms_ and SL_rms_. As for SPL_pp_ and SL_pp_, the SPL_rms_ and SL_rms_ values were averaged on 10 antennal rasps per individual. The SPL_rms_ and SL_rms_ values were used to estimate transmission loss (TL), as well as to compute the passive sonar equation to estimate detection ranges^42^.

We also calculated the dominant frequency (in kHz) of each antennal rasp, represented as the frequency where the PSD was maximal. At large distances, some sounds were lost in the ambient noise while looking at the time domain signals, their SPL_pp_ and SPL_rms_ were thus not computed. However, they were still visible in the frequency domain; in this case, their dominant frequencies were estimated.

### Ambient noise characterization

Recordings of ambient noise (10 min each) from the 8 hydrophones were first visualized to ensure the absence of antennal rasps. Because anthropogenic noise affected the ambient noise recordings during the experiment, sound sequences were both cut into 20 sequences of 30 seconds each, and we randomly selected 3 of 20 sequences from each recording. The sequences where anthropogenic noise (mainly shipping noise) was dominant were not taken into account in the analysis. We calculated the SPL_rms_ of all selected 30 s long sequences. This SPL_rms_ was calculated over the same frequency band as the antennal rasp SPL_rms_ and SL_rms_ (10–78 kHz), except for data from the Wildlife recorder, for which SPL_rms_ was calculated over the 10–48 kHz frequency band. This provided a mean value for the ambient noise at each hydrophone placement, and was referred to the ambient noise level (ANL).

### Evaluation of transmission losses

The datasets of the averaged SPL_rms_ for each individual and for each distance were fitted with nonlinear least-squares regressions using custom-made scripts in MATLAB. We used the following equation^42^:

1 $${{\rm{SPL}}}_{{\rm{rms}}}={{\rm{SL}}}_{{\rm{rms}}}-{\rm{TL}}$$

where TL is the transmission loss (in dB). TL represents the loss of intensity due to the geometrical spreading of sounds in a physical medium^42^, and was calculated as the slope of the logarithmic regression between SPL_rms_ and the distance from the spiny lobsters, which was expressed as:

2 $${\rm{TL}}=a\times {\log }_{10}({\rm{r}})$$

where r is the distance between the spiny lobsters and the hydrophones (in meters), and *a* is the geometrical TL term.

We obtained 19 different TL models using this method on the dataset generated by each animal (i.e. known SPL_rms_, SL_rms_ and r). The measurements from the 5 very small individuals were not included in this analysis because we did not have enough measurement points as they were only detectable between up to 5 and 10 m (see Results). Moreover, a global TL coefficient was also estimated using a global dataset obtained by merging the sounds from the 19 spiny lobsters. We compared this global TL with other theoretical models of TL commonly used in the bioacoustic literature^19^. In theory, the spherical spreading loss (${\rm{TL}}=20\times {{\rm{\log }}}_{10}({\rm{r}})$) prevails near the source where sound propagates uniformly in all directions. The cylindrical spreading loss (${\rm{TL}}=10\times {{\rm{\log }}}_{10}({\rm{r}})$), prevails in shallow waters where sound cannot propagate as a spherical wave in all directions but only as a cylindrical wave bounded by the sea floor and the sea surface. We also used a combined TL, termed the ‘practical’ spreading loss (${\rm{TL}}=15\times {{\rm{\log }}}_{10}({\rm{r}})$), which occurs between the prediction of the two other spreading models described above^48^.

### Estimations of detection ranges

For the purpose of this study, we assumed that signal detection by hydrophones was primarily limited by the TL (previously calculated), the ANL and the absorption (α) of the high frequencies considering the detection ranges (i.e. kilometre scale).

Using the previous results, we estimated the detection ranges of the antennal rasps by resolving the passive sonar equation (in the frequency domain) for all 24 spiny lobsters^42^:

3 $${{\rm{SL}}}_{{\rm{rms}}}-{\rm{TL}}-{\rm{\alpha }}-{\rm{ANL}}={\rm{SNR}}$$

Where:

- SL_rms_ is the source level in dB re 1 µPa^2^ (in root-mean-square; averaged on 10 measurements per spiny lobster), calculated in the 10–78 kHz frequency band^42^.
- TL is the global coefficient of TL previously calculated for the 19 largest spiny lobsters. We also used the models of cylindrical and practical TL detailed above.
- α is the coefficient of attenuation, depending on the frequency of the sound. Here, it was used at the dominant frequency that was commonly found above 10 m in the recorded antennal rasps, which was estimated at 30 kHz (see Results). Thus, the coefficient of absorption was calculated to be 7 dB per km^49^.
- ANL is the ambient noise level in dB re 1 µPa^2^ (in root-mean-square) calculated over the same band of frequencies than the SL_rms_ (10–78 kHz). We used two different values of ANL. First, we reported the mean ANL recorded by our hydrophones during the study. Second, we used one theoretical (but still realistic) value of ANL based on Wenz curves and calculated with a wind speed of 5 knots in the same frequency band than the ANL *in situ*^33^.
- SNR is the signal-to-noise ratio which corresponds to the minimum threshold needed for the hydrophones to detect the sound above the ANL. We used two different SNRs of 5 and 10 dB widely accepted for sonar systems^42^.

Because our sound recordings were performed in shallow waters (<10 m), we considered the water column as non-stratified. Thus, the effects of sound speed were assumed to be independent of depth^4^, and were not taken into account in this equation. Also note that in theory, the absorption coefficient α should be embedded into our estimated TL model. However, the impact of α is relatively small over our array range, with a loss smaller than 1 dB, which is negligible with respect to the geometrical TL. On the other hand, at larger ranges, the impact of α becomes important. We thus decided to add α in Eq. (3); this ensures that detection ranges are not over estimated.

### Statistical analysis

We examined the correlations between SL_pp_ and TL with body size using Pearson tests (α = 0.05). Analysis were performed using R version 3.5.1^50^.

### Ethical statement

Experiments with European spiny lobsters are not subject to restriction for animal scientific research according to the French legislation and the European Community Council Directive of September 2010 (2010/63/UE). However, we followed the ARRIVE guidelines^51^ to ensure that all experiments were performed under good conditions. Animals were handled with care during the study and their health status were checked daily by the authors. No specimens were harmed during this study and there was no mortality. At the end of the study, 7 adults were kept in the laboratory for other experiments. All the other animals were released back into the environment where they were collected.

## Data Availability

The codes developed in Matlab for signal processing can be provided if requested by the reviewers. We can also communicate.wav files of antennal rasps recorded during the *in situ* experiment.

## References

[1] Tyack, P. L., & Clark, C. W. [Communication and acoustic behavior of dolphins and whales] *Hearing by Whales and Dolphins* [Au, W. W. L., Popper, A. N., & Fay, R. R. (eds.)] 156–224 (Springer-Verlag, New York, 2000).

[2] Lobel, P. S., Kaatz, I. M., & Rice, A. N. [Acoustical behavior of coral reef fishes] *Reproduction and Sexuality in Marine Fishes: Patterns and Processes* [Cole, K. S. (eds.)] 307–386 (University of California Press, 2010).

[3] Tricas T, Boyle K (2014). Acoustic behaviors in Hawaiian coral reef fish communities. Mar. Ecol. Progr. Ser..

[4] Urick, R. J. *Principles of Underwater Sound* 423 (McGraw-Hill, 1983).

[5] Stafford KM, Fox CG, Clark DS (1998). Long-range acoustic detection and localization of blue whale calls in the northeast Pacific Ocean. J. Acoust. Soc. Am..

[6] Zimmer WM, Harwood J, Tyack PL, Johnson MP, Madsen PT (2008). Passive acoustic detection of deep-diving beaked whales. J. Acoust. Soc. Am..

[7] Bonnel J, Thode AM, Blackwell SB, Kim K, Macrander AM (2014). Range estimation of bowhead whale (*Balaena mysticetus*) calls in the Arctic using a single hydrophone. J. Acoust. Soc. Am..

[8] Mann DA, Lobel PS (1997). Propagation of damselfish (Pomacentridae) courtship sounds. J. Acoust. Soc. Am..

[9] Alves D, Amorim MCP, Fonseca PJ (2016). Assessing acoustic communication active space in the Lusitanian toadfish. J. Exp. Biol..

[10] Sprague MW, Luczkovich JJ (2004). Measurement of an individual silver perch *Bairdiella chrysoura* sound pressure level in a field recording. J. Acoust. Soc. Am..

[11] Locascio JV, Mann DA (2011). Localization and source level estimates of black drum (*Pogonias cromis*). J. Acoust. Soc. Am..

[12] Schmitz, B. [Sound production in Crustacea with special reference to the Alpheidae] *The crustacean nervous system* [Wiese, K. (eds.)] 536–547 (Springer, New York, 2002).

[13] Jézéquel Y, Bonnel J, Coston-Guarini J, Guarini JM, Chauvaud L (2018). Sound characterization of the European lobster *Homarus gammarus* in tanks. Aquat. Biol..

[14] Jézéquel Y, Coston-Guarini J, Chauvaud L, Bonnel J (2020). Acoustic behaviour of male European lobsters (*Homarus gammarus*) during agonistic encounters. J. Exp. Biol..

[15] Jézéquel Y, Bonnel J, Coston-Guarini J, Chauvaud L (2019). Revisiting the bioacoustics of European spiny lobsters *Palinurus elephas*: comparison of antennal rasps in tanks and *in situ*. Mar. Ecol. Prog. Ser..

[16] Meyer-Rochow VB, Penrose JD (1976). Sound production by the western rock lobster *Panulirus longipes* (Milne Edwards). J. Exp. Mar. Biol. Ecol..

[17] Au WWL, Banks K (1998). The acoustics of the snapping shrimp *Synalpheus parneomeris* in Kaneohe Bay. J. Acoust. Soc. Am..

[18] Rogers, P. H., & Cox, M. [Underwater sounds as a biological stimulus] *Sensory Biology of Aquatic Animals* [Atema, J., Fay R. R, Popper, A. N., & Tavolga, W. N. (eds.) 131–149 (Springer-Verlag, New York, 1988).

[19] Bass, A. H., & Clark, C. W. [The physical acoustics of underwater sound communication] *Acoustic Communication* [Simmons, A. M., Popper, A.N., & Fay, R. R. (eds.)] 15–64 (Springer-Verlag, New York, 2003).

[20] Richardson, W. J., Greene, C. R. J., Malme, C. I., & Thomson, D. H. *Marine Mammals and Noise* (Academic Press, London, 1995).

[21] Radford CA, Tindle CT, Montgomery JC, Jeffs AG (2011). Modelling a reef as an extended sound source increases the predicted range at which reef noise may be heard by fish larvae. Mar. Ecol. Prog. Ser..

[22] Butler J, Butler MJ, Gaff H (2017). Snap, crackle, and pop: Acoustic-based model estimation of snapping shrimp populations in healthy and degraded hard-bottom habitats. Ecol. Indic..

[23] Coquereau L (2016). Sound production and associated behaviours of benthic invertebrates from a coastal habitat in the north-east Atlantic. Mar. Biol..

[24] Madsen PT, Wahlberg M (2007). Recording and quantification of ultrasonic echolocation clicks from free-ranging toothed whales. Deep-Sea Res. (1 Oceanogr. Res. Pap.).

[25] Patek SN, Shipp LE, Staaterman ER (2009). The acoustics and acoustic behavior of the California spiny lobster (*Panulirus interruptus*). J. Acoust. Soc. Am..

[26] Patek SN (2001). Spiny lobsters stick and slip to make sound. Nature.

[27] Sanborn AF, Phillips PK (1995). Scaling of sound pressure level and body size in cicadas (Homoptera: Cicadidae; Tibicinidae). Ann. Entomol. Soc. Am..

[28] Bennet-Clark HC (1998). Size and scale effects as constraints in insect sound communication. Philos. Trans. R. Soc. Lond., B, Biol. Sci..

[29] Goñi R, Latrouite D (2005). Review of the biology, ecology and fisheries of Palinurus species of European waters: *Palinurus elephas* (Fabricius, 1787) and *Palinurus mauritanicus* (Gruvel, 1911). Cah. Biol. Mar..

[30] Goñi, R. Palinurus elephas. The IUCN Red List of Threatened Species, e.T169975A1281221 (2014).

[31] Kikuchi M, Akamatsu T, Takase T (2015). Passive acoustic monitoring of Japanese spiny lobster stridulating sounds. Fish. Sci..

[32] Herrnkind, W. F. [Spiny lobsters: patterns of movement] *Biology and Management of Lobsters. Vol. 1. Physiology and Behavior* [Cobb, J. S., & Phillips, B. F. (eds.)] 349–407 (Academic Press, New York, 1980).

[33] Wenz GM (1962). Acoustic ambient noise in ocean spectra and sources. J. Acoust. Soc. Am..

[34] Kaplan MB, Mooney TA (2016). Coral reef soundscapes may not be detectable far from the reef. Sci. Rep..

[35] Parks SE (2011). Sound production behavior of individual North Atlantic right whales: implications for passive acoustic monitoring. Endanger. Species Res..

[36] Locascio JV, Mann DA (2008). Diel periodicity of fish sound production in Charlotte Harbor, Florida. Trans. Am. Fish. Soc..

[37] Popper AN, Salmon M, Horch KW (2001). Acoustic detection and communication by decapod crustaceans. J. Comp. Physiol..

[38] Popper AN, Hawkins AD (2018). The importance of particle motion to fishes and invertebrates. J. Acoust. Soc. Am..

[39] Hunter E (1999). Biology of the European spiny lobster *Palinurus elephas* (Fabricius, 1787) (Decapoda, Palinuridea). Crustaceana.

[40] Ceccaldi, H. J., & Latrouite, D. [The French fisheries for the European spiny lobster *Palinurus elephas*] *Spiny lobster fisheries and culture* [Phillips, B. F., & Kittaka, J. (eds.)] 200–209 (Blackwell, Oxford, 2000)

[41] Díaz D, Marí M, Abelló P, Demestre M (2001). Settlement and juvenile habitat of the European spiny lobster *Palinurus elephas* (Crustacea: Decapoda: Palinuridae) in the western Mediterranean Sea. Sci. Mar..

[42] Ainslie, M. A. *Principles of sonar performance modelling* 800 (Springer, Berlin, 2010).

[43] Goodall, C., Chapman, C., & Neil, D. [The acoustic response threshold of the Norway lobster, *Nephrops norvegicus* (L.) in a free sound field] Frontiers in crustacean neurobiology [Wiese, K., -Krenz, W. D., Tautz, J., Reichert, H., & Mulloney, B. (eds.)] 106−11 (Springer-Basel, Berlin, 1990).

[44] Rountree RA (2006). Listening to fish: applications of passive acoustics to fisheries science. Fisheries.

[45] Mann, D. A., Hawkins, A. D., & Jech, J. M. [Active and passive acoustics to locate and study fish] *Fish bioacoustics* [Webb, J. F., Fay, R. R., & Popper, A. N. (eds.)] 279–309 (Springer, New York, 2008).

[46] Audacity Team Audacity®. version 2.1.1. www.audacity team.org (accessed 14 June 2019) (2015).

[47] Madsen PT (2005). Marine mammals and noise: Problems with root mean square sound pressure levels for transients. J. Acoust. Soc. Am..

[48] Coates, R. F. W. *Underwater acoustic systems* 188 (Wiley, New York, 1989).

[49] Fisher FH, Simmons VP (1977). Sound absorption in sea water. J. Acoust. Soc. Am..

[50] R Core Team. R: a language and environment for statistical computing (R Foundation for Statistical Computing, Vienna, 2018).

[51] Kilkenny C, Browne WJ, Cuthill IC, Emerson M, Altman DG (2010). Improving bioscience research reporting: the ARRIVE guidelines for reporting animal research. PLoS Biol..

